# Idiotype vaccines produced with a non-cytopathic alphavirus self-amplifying RNA vector induce antitumor responses in a murine model of B-cell lymphoma

**DOI:** 10.1038/s41598-021-00787-5

**Published:** 2021-11-02

**Authors:** Erkuden Casales, Eva Martisova, Helena Villanueva, Ascensión López Díaz de Cerio, Susana Inoges, Noelia Silva-Pilipich, María Cristina Ballesteros-Briones, Alejandro Aranda, Jaione Bezunartea, Maurizio Bendandi, Fernando Pastor, Cristian Smerdou

**Affiliations:** 1 Division of Gene Therapy and Regulation of Gene Expression, Cima Universidad de Navarra, Av. Pio XII 55, 31008 Pamplona, Spain; 2 Instituto de Investigación Sanitaria de Navarra (IdISNA), Pamplona, Spain; 3 Aptamer Platform, Molecular Therapeutics Program, Cima Universidad de Navarra, Pamplona, Spain; 4 Department of Hematology and Cell Therapy Area, Department of Immunology and Immunotherapy, University Clinic of Navarra, Pamplona, Spain; 5 Present Address: Section On Hematology/Oncology, Wake Forest University Comprehensive Cancer Center, Winston-Salem, NC USA; 6 Present Address: Section of Hematology/Oncology, VA Medical Center, Salisbury, NC USA

**Keywords:** Protein vaccines, Expression systems, Cancer immunotherapy

## Abstract

A promising therapy for patients with B-cell lymphoma is based on vaccination with idiotype monoclonal antibodies (mAbs). Since idiotypes are different in each tumor, a personalized vaccine has to be produced for each patient. Expression of immunoglobulins with appropriate post-translational modifications for human use often requires the use of stable mammalian cells that can be scaled-up to reach the desired level of production. We have used a noncytopathic self-amplifying RNA vector derived from Semliki Forest virus (ncSFV) to generate BHK cell lines expressing murine follicular lymphoma-derived idiotype A20 mAb. ncSFV/BHK cell lines expressed approximately 2 mg/L/24 h of A20 mAb with proper quaternary structure and a glycosylation pattern similar to that of A20 mAb produced by hybridoma cells. A20 mAb purified from the supernatant of a ncSFV cell line, or from the hybridoma, was conjugated to keyhole limpet hemocyanin and used to immunize Balb/c mice by administration of four weekly doses of 25 µg of mAb. Both idiotype mAbs were able to induce a similar antitumor protection and longer survival compared to non-immunized mice. These results indicate that the ncSFV RNA vector could represent a quick and efficient system to produce patient-specific idiotypes with potential application as lymphoma vaccines.

## Introduction

The idiotype (Id) of a B-cell lymphoma refers to the unique amino acid sequences of the variable fragments of the heavy and light chains of the surface immunoglobulin expressed by tumor cells. For this reason, the Id represents a patient- and tumor-specific antigen with great potential for lymphoma-targeted therapy^1,2^. Passive immunotherapy using anti-Id monoclonal antibodies (mAbs) has been shown to generate clinical responses, although relapse is likely to occur due to the emergence of tumor escape mutants^3,4^. In this regard, the use of polyclonal anti-Id antibodies could prevent the emergence of tumor escapees, however, they are more difficult to generate^5^.

Id vaccination is an active type of immunotherapy that aims to elicit a polyclonal Id-specific immune response. Both preclinical and clinical studies have shown that Id vaccination has the potential to elicit cellular and humoral immune responses against B-cell lymphomas^1^. Different strategies for vaccination can be employed, including administration of Id as recombinant protein, cellular vaccines, or in vivo gene therapy^2,6,7^.

Vaccination using protein is hindered by the need to produce the Id protein for each patient, which can be time-consuming and expensive. Traditionally, Id protein production was carried out using hybridomas. More recently, recombinant technology has allowed to shorten vaccine production times. In this approach, the sequences of both heavy and light chain variable regions of the Id are cloned into an expression vector for the production of Id protein in different systems, such as mammalian cells, insect cells, tobacco plants, and bacteria^1^. As the Id is a self-antigen, its low immunogenicity must be taken into account when designing vaccination protocols to achieve an effective immune response. To enhance its immunostimulatory properties, the Id is usually conjugated to an immunogenic carrier, typically keyhole limpet hemocyanin (KLH), and administered alongside an adjuvant such as granulocyte–macrophage colony-stimulator factor (GM-CSF). Several proof-of-principle studies carried out in a limited number of patients have been successful at providing evidence of biological and clinical efficacy of this type of immunotherapy^8^. Nevertheless, large-scale randomized clinical trials have failed so far to show a significant difference between control treatment and Id vaccination, although important flaws in the study designs may have compromised their results^2,9^.

Mammalian cells are usually employed for Id expression as they provide the appropriate post-translational modifications. Self-replicating RNA vectors, such as those derived from alphaviruses, have shown a high efficacy for protein expression in mammalian cells. These vectors include Sindbis virus (SIN), Semliki Forest Virus (SFV) and Venezuelan equine encephalitis virus (VEEV) and are usually based on RNA replicons in which the viral structural genes have been substituted by a heterologous gene. They provide high and rapid protein expression, although in a transient way, due to the cytopathic effect of viral RNA replication in most mammalian cells^10^. To overcome this drawback, several mutations have been described that make these vectors less cytopathic, allowing prolonged protein expression in vitro^11,12^. Previously, we have reported a noncytopathic mutant derived from SFV (ncSFV) that contains mutations P718T and R649H in the nsp2 subunit of the replicase. This ncSFV generates high expression levels of heterologous proteins in vitro, similar to wild type SFV^13^. By including a puromycin resistance cassette (pac) in ncSFV, stable cell lines expressing high levels of heterologous proteins can be easily obtained through selection with this antibiotic^14^.

In this work, BHK stable cell lines expressing the Id from murine A20 B cell lymphoma were generated using the ncSFV RNA vector. A20 Id was purified from the supernatant of a high expressing ncSFV cell line and used to immunize mice. When challenged with A20 cells, immunized mice showed a significant reduction of tumor size and increase in survival, compared to the control group.

## Materials and methods

### Cells

BHK-21 cells (ATCC: CCL-10) and derived stable cell lines were cultured in BHK-21 Glasgow MEM (Gibco BRL, UK) supplemented with 5% FCS, 10% tryptose phosphate broth, 2 mM glutamine, 20 mM HEPES, 100 µg/ml streptomycin and 100 IU/ml penicillin (BHK complete medium). A20 lymphoma cells (ATCC^®^ TIB-208) were cultured in RPMI medium supplemented with 10% FCS, glutamine and streptomycin/penicillin as previously described for BHK cells. Hybridoma cells were obtained by fusion of A20 cells with P3X63Ag8.653 (ATCC^®^ CRL-1580™) as described^15^.

### Plasmid constructs

**Figure 1:**
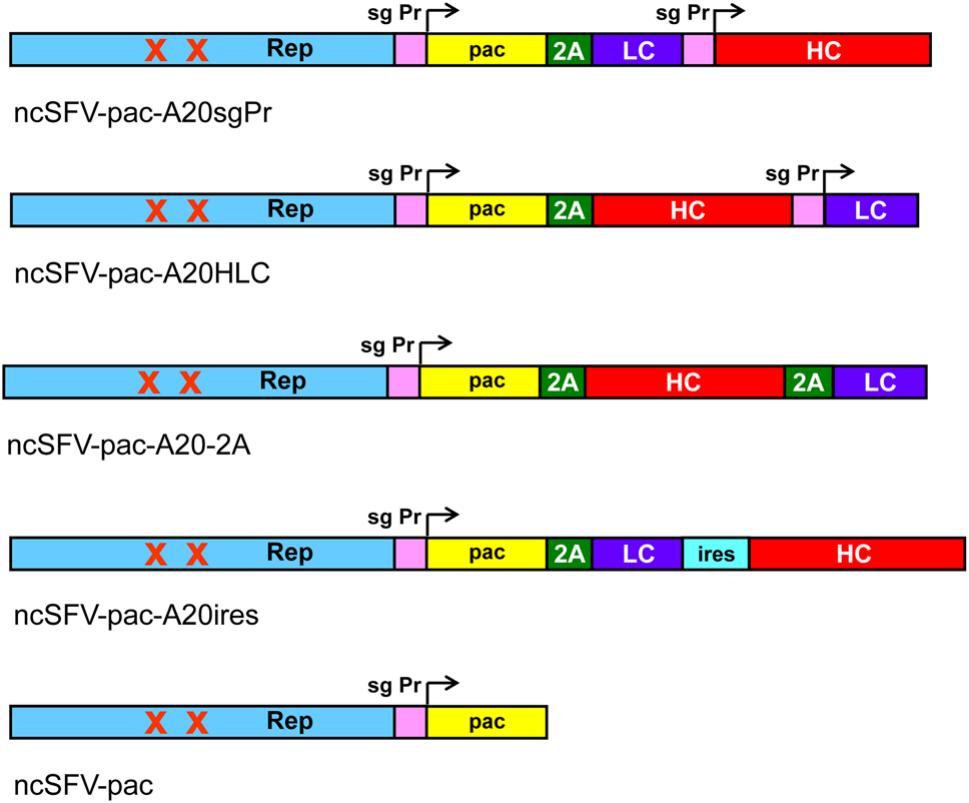
Schematic representation of vectors expressing murine follicular lymphoma-derived A20 mAb. Noncytopathic vectors (ncSFV-pac) contain mutations P718T and R649H in the nsp2 subunit of the replicase (Rep), indicated by two Xs, and express the pac gene downstream of the viral subgenomic promoter (sg Pr). The sequence of the small FMDV 2A autoprotease (2A) was used to fuse the pac gene to genes coding for the heavy (HC) or light chain (LC) of A20 mAb. A second sg Pr, the 2A sequence, or the internal ribosome entry site (IRES) from encephalomyocarditis virus were used to express separately HC and LC as indicated in the diagrams.

Plasmid ncSFV-pac2A, which contains the P718T and R649H mutations in the nsp2 subunit of the SFV replicase and the pac gene downstream of the subgenomic promoter (sgPr) followed by the sequence coding for the foot and mouth disease virus 2A autoprotease (2A), was previously described by our group^13,14^ and used to generate ncSFV-pac plasmids expressing A20 mAb. For this purpose we first purifed total RNA from A20 lymphoma cells and synthesized cDNA using random primers. Since the sequence of the A20 mAb was already known^7^, we designed specific oligonucleotides to amplify the HC and LC sequences (Supplementary Table 1) from the obtained cDNA. We generated DNA fragments containing both the HC and LC chains of the antibody by PCR and subcloned them into the Xma I site of the ncSFV-pac2A plasmid (Fig. 1). PCR fragments were obtained as follows: (i) For ncSFV-pacA20sgPr, a crossover PCR was designed to insert an sgPr sequence between both chains. The first PCR was performed with oligonucleotides 1 + 2 (Supplementary Table 1) and the second PCR with oligonucleotides 3 + 4. Fragments obtained from both PCRs were purified, mixed in equal amounts and used as template for a crossover PCR using oligonucleotides 1 + 4, obtaining a 2191 bp fragment with the LC followed by sgPr and HC. This fragment was subcloned into pGEM-T, sequenced, digested with Xma I and subcloned into ncSFV-pac, using the same restriction site. (ii) For ncSFV-pac-A20HLC a similar strategy was followed using primers 5 + 6 and 7 + 8 to generate two independent fragments and primers 5 + 8 for the crossover PCR, generating a 2196 bp fragment with the HC followed by sgPr and LC. (iii) For ncSFV-pac-A20-2A a similar strategy was used using primers 5 + 9 and 10 + 8 to generate two independent fragments and primers 5 + 8 for the crossover PCR, generating a 2193 bp fragment with the HC followed by 2A sequence and LC. (iv) Finally, to generate ncSFV-pac-A20ires, we first used a similar strategy using primers 1 + 11 and primers 12 + 13 to generate two independent DNA fragments and primers 1 + 13 (in this case using as template a plasmid containing the IRES sequence^16^ for crossover PCR, obtaining a construct with the LC fused to the internal ribosome entry site (IRES) from encephalomyocarditis virus, which was cloned into pGEM-T (pLC-IRES). Another PCR was performed with primers 4 + 14 to generate a fragment with the HC sequence that was subcloned into pLC-IRES generating pLC-IRES-HC. From this intermediate plasmid a 2826 pb fragment with the LC followed by the IRES sequence and HC was extracted with Xma I and sucbloned into ncSFV-pac2A. Plasmid pSFV-S2-9-pac, referred here as ncSFV-pac was used to generate control cell lines^14^.

### Selection of cell lines with ncSFVpac vectors

SFV-derived plasmids were linearized by digestion with SpeI and transcribed in the presence of cap analog (New England Biolabs, Ipswich, MA) using SP6 polymerase (New England Biolabs)^17^. Fifty µg of in vitro transcribed RNA were electroporated into 5 × 10^6^ BHK-21 cells by electroporation as described previously^13^. After transfection, cells were allowed to recover for 24–28 h before addition of puromycin at 5 µg/ml (Sigma, St. Louis, MO). To select for puromycin resistant cells, medium was replaced every 2–3 days with fresh puromycin containing medium. Upon selection, cells were always passaged in the presence of puromycin at the indicated concentration.

### Analysis of protein expression

The amount of A20 mAb present in supernatants from cells transfected with ncSFV-pac-A20 vectors was quantified by a total mouse IgG ELISA kit (Mabtech, Sweden) using as standard A20 mAb purified from the A20 hybridoma cell line. This standard mAb was previously quantified by Bradford analysis. For Western blot experiments, supernatants from cells transfected with SFV vectors expressing A20 mAb were analyzed under reducing (with dithiothreitol, DTT) and non-reducing (without DTT) conditions in 8% or 12% polyacrylamide gels, respectively, incubating with a polyclonal goat antibody specific for mouse IgG conjugated with horseradish peroxidase (Sigma, St. Louis, MO). Cell lysates were also analyzed with rabbit polyclonal antisera specific for SFV nsp2^13^ or for actin (Sigma). Proteins were visualized using the Western Lightning Chemiluminiscence Reagent Plus (Perkin Elmer Life Sciences, Waltham, MA).

### Purification of A20 mAb

A cell line selected with ncSFV-pac-A20sgPr vector was grown to a final quantity of approximately 2 × 10^8^ cells, corresponding to six consecutive passages. Cells were first incubated with 500 ml EX-CELL™ CD CHO medium without FBS (Sigma) during 36 h at 33 °C. This medium was collected and then cells were incubated again with 500 ml of new medium for 24 h at the same temperature. Culture media from the two incubations were pooled and centrifuged at 1000*g* for 5 min. Recombinant mAb was purified by Protein A Sepharose column (HiTrap Protein A HP, GE Healthcare Bio-Sciences, Pittsburgh, PA) at 4 °C and at a constant speed of 1 ml/min during approximately 16 h using an ÄKTA protein purification system (GE Healthcare Bio-Sciences). The mAb was eluted with 100 mM glycine (pH 3). The presence of the mAb was analyzed at each purification step by SDS-PAGE followed by Coomassie staining or Western blot as described earlier. The purified mAb was gel digested with trypsin and protein identification was obtained by analysis of the digests in a coupled liquid chromatography and tandem mass spectrometry (LC–MS/MS). A20 mAb produced by the hybridoma cell line had been previously purified using a similar procedure.

### Analysis of A20 glycosylation

For glycosylation analysis, samples of purified mAb were treated with N-glycosidase (Calbiochem, San Diego, CA) according to the manufacturer’s instructions. Briefly, samples were heated at 100 °C for 5 min, cooled to room temperature, and incubated with glycosidase during 3 h at 37 °C in the presence of 0.75% Triton X-100. After treatment, samples were analyzed by Western blot as described earlier.

### Vaccination studies

Purified A20 mAb was first conjugated to Keyhole Limpet Hemocyanin (KLH, Biosyn Corporation, Carlsbad, CA) as previously described^15^. Four-week-old Balb/c female mice (Envigo, Spain) were randomly distributed in three groups that were immunized with A20 mAb purified from hybridoma or ncSFV-pac-A20sgPr cells, or with PBS. In the first two cases, mice received four subcutaneous immunizations, given once a week, of 25 µg of purified mAb conjugated with the same amount of KLH in combination with 10,000 U of GM-CSF (BD Pharmingen) in a total volume of 50 µl. Control mice received the same volume of PBS. In addition, at 24 h, 48 h, and 72 h after each A20 mAb vaccination, mice received 10,000 U of GM-CSF diluted in 50 µl of PBS which were administered subcutaneously close to the site of the vaccine injection (Fig. 5A). All mice were retroorbitally bled before the first vaccination and 10 days after the last vaccine boost. Fifteen days after the last vaccine dose all mice were subcutaneously injected with 2 × 10^5^ A20 lymphoma cells and tumor growth was determined at different time points by measuring two perpendicular diameters. Tumor volume was calculated with the formula: d^2^ × D/2 where d = smaller diameter; D = larger diameter. One mouse out of 18 and one mouse out of 22 were excluded from the A20-SFV and PBS groups in Fig. 5A, since they were detected as outlayers using Prism software (GraphPad Software, San Diego, CA). Animal studies were approved by the Universidad de Navarra ethical committee (study 063/14-E44/15) for animal experimentation under Spanish regulations. This study was carried out in compliance with the ARRIVE guidelines.

### A20 specific ELISA

ELISA plates were coated with purified A20 mAb at a concentration of 10 µg/ml. After blocking with PBS containing 1% fat-free milk, plates were incubated with serial dilutions of mice sera (in PBS with 1% fat free milk). Finally, plates were incubated with horseradish peroxidase-conjugated anti mouse IgG1 (Pharmingen).

### Statistical analyses

Graphpad Prism software (version 9.2.0) was used for statistical analysis. Survival of tumor-bearing animals is represented by Kaplan–Meier plots and was analyzed by log-rank test. To compare tumor size in experimental groups, the Kruskal–Wallis test, followed by the Dunn multiple-comparison test, was used for nonparametric data.

## Results

### Generation and characterization of SFV-based stable cell lines expressing idiotype antibody A20

In order to generate mammalian stable cell lines expressing the Id A20, we cloned the genes coding for its heavy (HC) and light chains (LC) into ncSFV-pac vector as described in “Materials and methods”. This RNA vector contains mutations P718T and R649H in the nsp2 subunit of the SFV viral replicase and expresses puromycin N-acetyl-transferase gene (pac) downstream of the viral subgenomic promoter, which allows selection of transfected cells in the presence of puromycin. Since efficient production of the mAb will require an optimal ratio of LC:HC^18^, we cloned their coding genes into ncSFV-pac using four different configurations, generating the following vectors: (i) ncSFV-pac-A20sgPr, in which HC is followed by LC using independent viral subgenomic promoters, (ii) ncSFV-pac-A20HLC, in which LC is followed by HC using independent viral subgenomic promoters, (iii) ncSFV-pac-A20-2A, in which HC is fused to LC using the foot and mouth disease virus 2A autoprotease sequence as a linker, and (iv) ncSFV-pac-A20ires, having LC and HC separated by an IRES sequence (Fig. 1).

**Figure 2:**
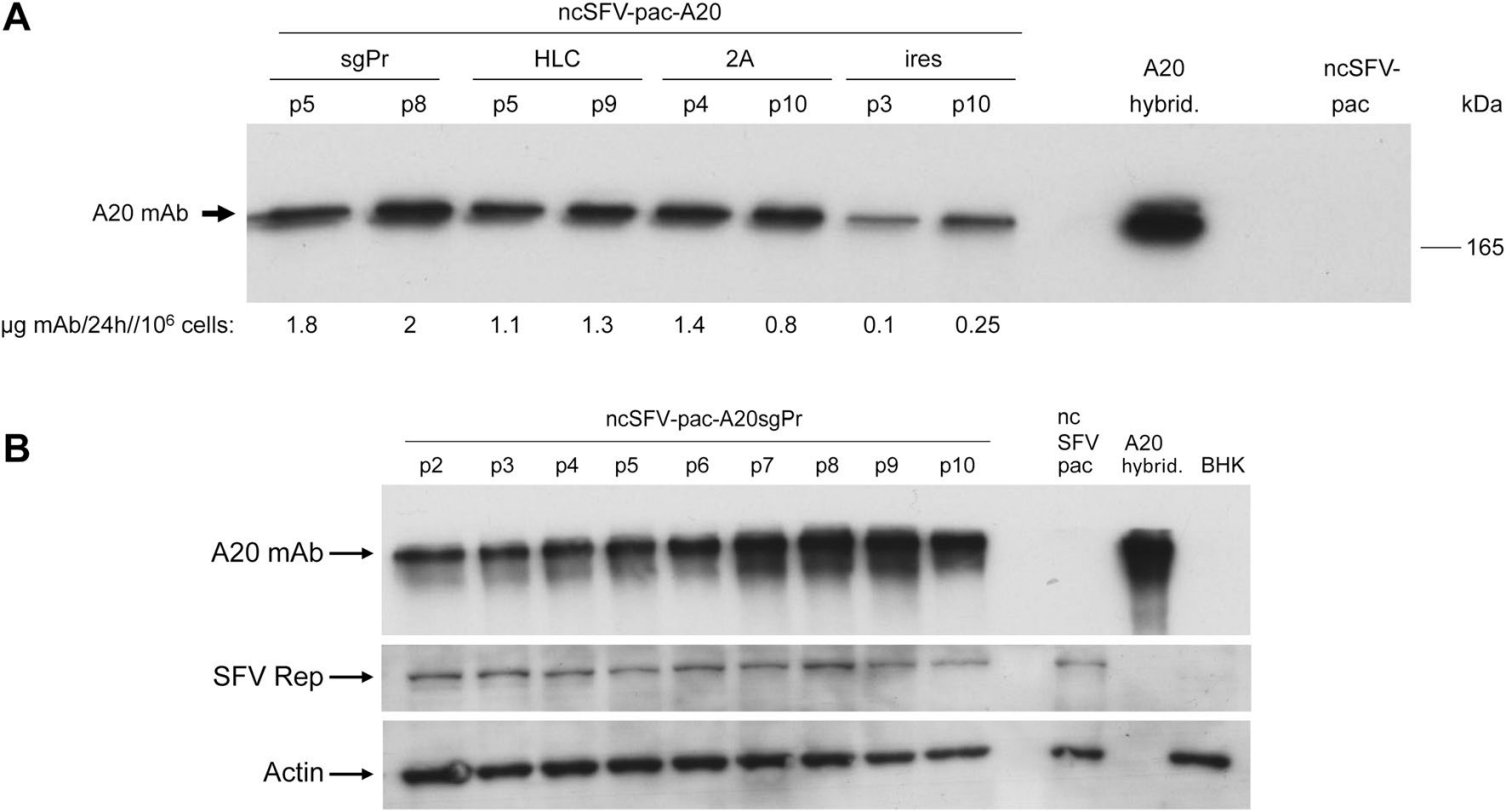
Analysis of A20 mAb expression in supernatants of stable BHK cell lines generated with ncSFV vectors. BHK cells were electroporated with ncSFV-pac2A RNA vectors and cell lines were selected in the presence of puromycin at 5 µg/ml. Selected cells were passaged ten times every 2 to 3 days with puromycin and the A20 mAb produced during 24 h in the supernatant of each passage was analysed by Western blot and specific ELISA. (**A**) Analysis of supernatants of two different passages (p) of a representative cell line obtained with each of the indicated ncSFV-pac vectors. Samples were run under non-reducing conditions and analyzed by Western blot with an anti mouse IgG antibody. The mAb expression level of each cell line was determined by specific ELISA and is indicated under the gel. A20 hybrid., 25 ng of A20 mAb purified from the A20 hybridoma cell line used as positive control. (**B**) Analysis of the stability of one representative cell line generated with ncSFV-pac-A20sgPr RNA. Selected cells were passaged 10 times and 6 µl of supernatant from each passage were used to analyze expression by Western blot as described in A (upper gel). SFV replicase and actin (used as a loading control) were also analyzed in cell extracts from each passage with specific antibodies (lower gel). BHK, untransfected BHK cells.

RNA was synthesized in vitro from each ncSFV-pac2A-A20 vector, or from control ncSFV-pac empty vector, and electroporated into BHK cells. Twenty-four hours after electroporation, puromycin was added at 5 µg/ml. When selected cells reached confluency, they were passaged ten times in the presence of the antibiotic during a period of 30 days. The expression of recombinant mAb was analyzed after each passage by Western blot and specific ELISA in the supernatants of at least two independent selected cell lines obtained for each vector. This analysis showed that all SFV-based cell lines expressed an mAb of similar size to the one expressed by the A20 hybridoma (Fig. 2A and Supplementary Fig. 1). In addition, expression of the A20 mAb was quite stable in all cell lines, with similar expression levels at early and late passages (Fig. 2A). The highest expression levels were obtained with vector ncSFV-pac-A20sgPr, reaching approximately 2 mg/10^6^ cells/24 h. Expression levels were approximately 50% lower in cells lines generated with ncSFV-pac-A20HLC and ncSFV-pac-A20-2A vectors, and between 10- and 20-fold lower in the case of ncSFV-pac-A20ires cell lines. In this last case we observed a strong reduction in the expression of HC compared to LC, which could explain the lower mAb production in these cell lines (Supplementary Fig. 1). We selected a ncSFV-pac-A20sgPr cell line for A20 mAb production, since it showed the highest expression levels and a very good level of stability (Fig. 2B).

### Purification and characterization of A20 Id

**Figure 3:**
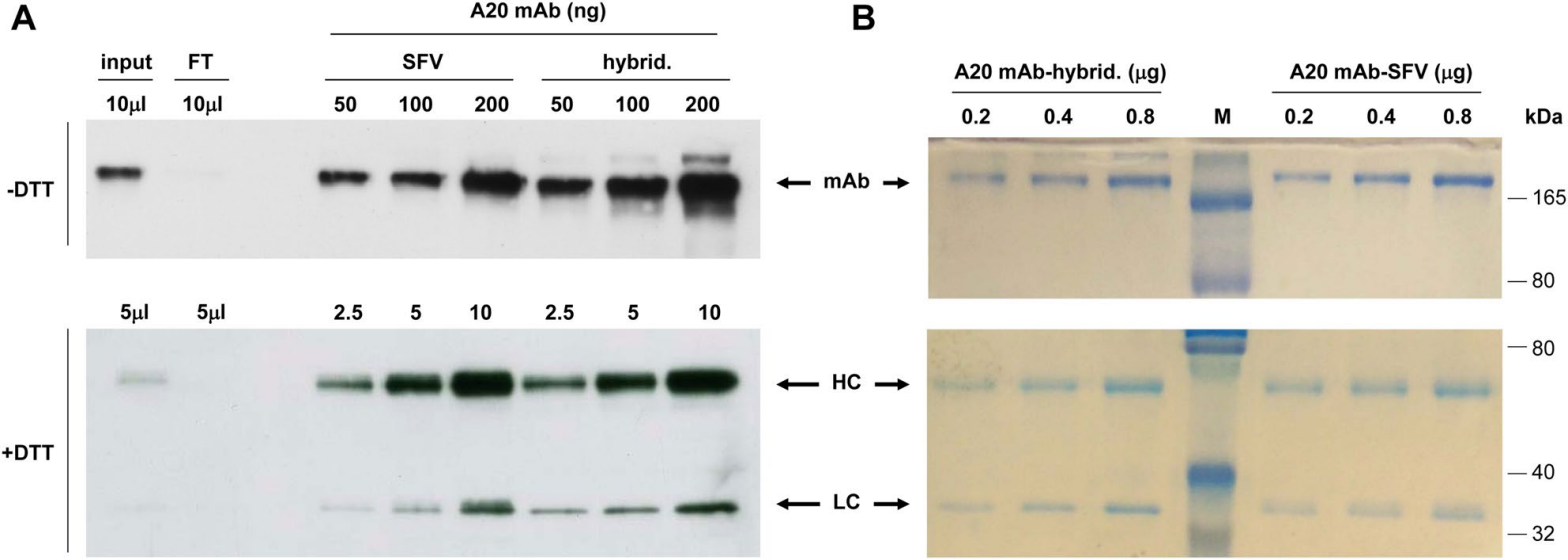
Purification of A20 mAb. Approximately 2 × 10^8^ BHK cells harbouring ncSFV-A20-sgPr vector were incubated during a total of 60 h at 33 °C and secreted A20 mAb was purified from 1L of supernatant by affinity chromatography on a protein A-Sepharose column. Purified A20 mAb (A20-mAb-SFV) was analysed by Western blot (**A**) and colloidal blue stainining (**B**) in parallel with mAb purified from hybridoma A20 cell line (A20-mAb-hybrid.) under non-reducing (-DTT) and reducing (DTT) conditions. Input, supernatant loaded into the column; FT, column flow-through.

To purify the A20 Id, a cell line selected with ncSFV-pac-A20sgPr was grown to a final quantity of approximately 2 × 10^8^ cells, corresponding to six consecutive passages. Cells were first incubated with 500 ml of medium without serum during 36 h. This medium was collected and then cells were incubated again with 500 ml of new medium for 24 h. Both supernatants were pooled and the mAb concentration was measured by ELISA, being approximately 1.75 µg/ml. The A20 Id was then purified using a Protein A Sepharose column as described in “Materials and methods”. Most of the mAb was retained in the column, since no antibody signal could be detected by Western blot in the flow through (Fig. 3A). The A20 Id was then eluted with 100 mM glycine (pH 3), obtaining a single elution peak as determined by UV absorbance. A yield of 1.6 mg/L of pure mAb was obtained from supernatants of ncSFV-pac-A20sgPr with a recovery of > 90%. Following a similar procedure, we purified the A20 Id from the supernatant of A20 hybridoma cells.

### Characterization of A20 Id

**Figure 4:**
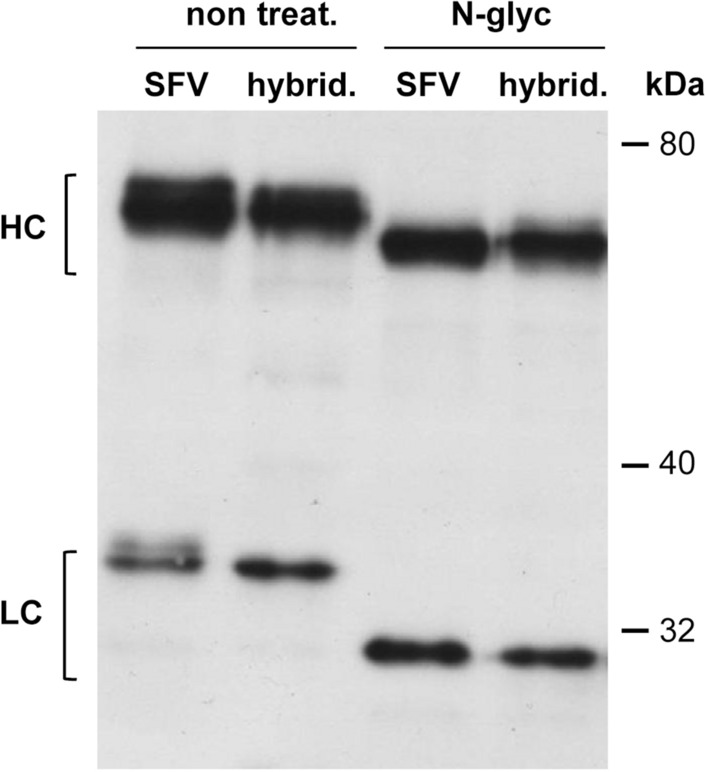
Analysis of glycosylation of A20 mAb. A20 mAb purified from ncSFV-A20-sgPr cell line (SFV) and from A20 hybridoma (hybrid.) were incubated with N-endoglycosidase (N-glyc) and analysed by Western blot under reducing conditions. Non-treated samples were used as control (non treat.).

Protein identity was confirmed by LC–MS/MS analysis for A20 Id purified from both ncSFV-pac-A20sgPr and hybridoma A20 cell lines. In both cases this technique allowed us to identify a great number of peptides corresponding to a IgG2a HC and a kappa LC, which are the type of chains present in A20 Id (data nor shown). The size and correct assembly of the mAb was verified by SDS-PAGE electrophoresis followed by Western blot with an anti mouse IgG antibody (Fig. 3A) and Coomassie staining (Fig. 3B). When samples containing different amounts of the mAbs were analysed in non-reducing conditions, a single high molecular weight (MW) band of > 165 kDa was detected in both analyses, corresponding to the fully assembled antibody (Fig. 3, upper gels). As expected, when the same samples were analyzed under reducing conditions, only bands corresponding to HC and LC chains were detected (Fig. 3 lower gels). The absence of other bands in the Coomassie analysis confirmed the high purity of the mAb (Fig. 3B). The apparent MW of the fully assembled mAb and the HC and LC chains was higher than expected (theoretical MW for mAb: 122.7 kDa, HC: 49 kDa, and LC: 12.38 kDa), which suggested a possible post-translational modification for both chains. To check if this higher molecular size was due to glycosylation, purified mAbs were treated with N-glycosidase as described in “Materials and methods” and analyzed by SDS-PAGE under reducing conditions. Removal of N-linked oligosaccharides increased the apparent electrophoretic mobility for both HC and LC, being more evident in the latter case (Fig. 4). This study showed that the A20 Id produced from the ncSFV-based BHK cell lines and the one produced from the hybridoma cell lines showed very similar characteristics.

### Vaccination of mice with idiotype antibody A20

**Figure 5:**
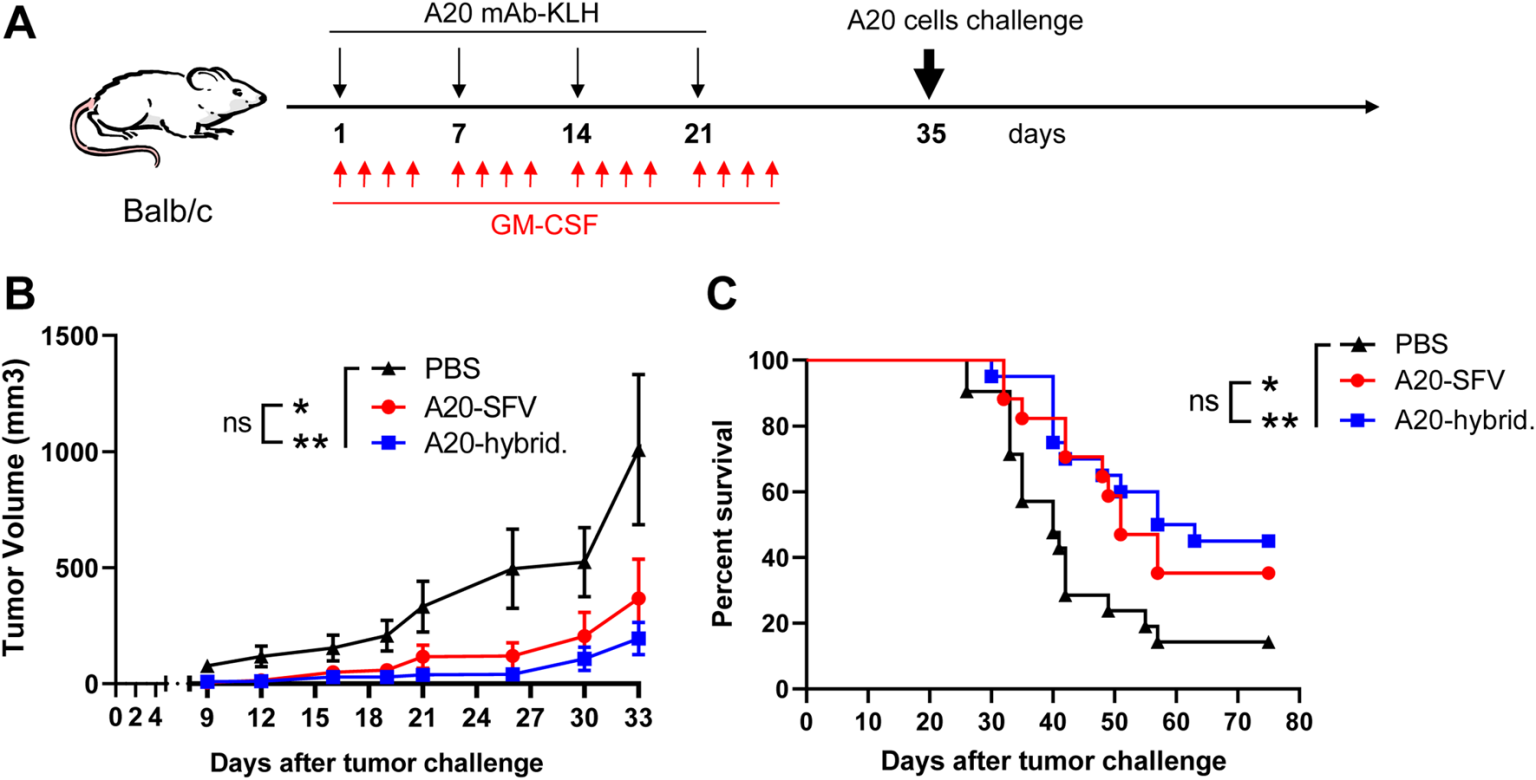
Vaccination of mice with purified A20. (**A**) Balb/c mice were vaccinated with A20 mAb purified from the supernatant of the ncSFV-A20-sgPr cell line (A20-SFV, n = 17) or from A20 hybridoma cells (A20-hybrid., n = 20) conjugated to KLH and in combination with GM-CSF as indicated in the diagram. Control mice received PBS (n = 21). Two weeks after the last immunization, mice were challenged with A20 tumor cells expressing A20 idiotype mAb. (**B**) Tumor growth in each treatment group. Data indicate mean ± SEM. The statistical analysis show comparison of tumor sizes at day 33 (**C**) Survival. Tumor-free mice surviving up to 75 days after tumor challenge were considered long-term survivors. *p < 0.05; **p < 0.01, ns, non significant. In B and C, graphs were generated with Graphpad Prism software (version 9.2.0) and correspond to pooled data of three independent experiments performed with three different batches of A20-SFV idiotype that produced similar results (tumor growth data for each individual experiment are shown in Supplementary Figure 3).

In order to check whether purified A20 mAbs could be used as Id vaccines, we first conjugated each antibody with KLH as described in “Materials and methods”. We then vaccinated a group of Balb/c mice with four subcutaneous doses of 25 µg of KLH-conjugated A20 Id produced in ncSFV-pac-A20sgPr cells, which were given once a week in combination with 1000 units of GM-CSF. Mice also received three additional doses of GM-CSF at 24 h, 48 h, and 72 h after each vaccination (see diagram in Fig. 5A). A second group of mice was vaccinated with KLH-conjugated A20 Id produced by A20 hybridoma cells following the same protocol. Finally, a control group was vaccinated with PBS. Animals were bled 10 days after the last vaccination and anti-A20 antibody levels were evaluated by specific ELISA (Supplementary Fig. 2). This analysis showed that mice vaccinated with the purified Id developed specific anti-Id antibodies, which had similar levels in mice receiving the A20 Id from ncSFV-based cell or hybridoma cells. Fifteen days after the last vaccine boost all mice were challenged subcutaneosly with A20 lymphoma cells and tumor size and survival were followed. As can be observed in Fig. 5B, vaccination of mice with purifed A20 Id resulted in a clear antitumor effect which was very similar in mice receiving the mAb from ncSFV-engineered cells or hybridoma cells. In addition, these two groups of mice showed a similar and significantly higher survival compared to control mice (Fig. 5C). These results demonstrate that the ncSFV system could be useful to produce personalized effective Id vaccines against lymphoma in an easy and quick way.

## Discussion

A very attractive approach to fight cancer is the possibility of inducing specific immune responses against tumor cells, leading to their destruction without affecting healthy tissues. Unfortunately, only a few tumor antigens have been identified that could be used as immunogens, or that could be targeted by mAbs or genetically modified T cells. However, a new area of research, that has expanded dramatically in recent years, has focused on the development of personalized therapeutic cancer vaccines based on the identification of neoantigens in tumor cells^19^. This strategy allows to design a specific vaccine for each patient, usually based on the administration of polypeptides containing several neoantigens with non-synonymous somatic mutations. The ultimate goal of these vaccines is the generation of antigen-specific CD8+ T cells able to recognize and destroy tumor cells.

A particular case of specific tumor antigen is the Id present in B-cell lymphomas. Ids are specific for each patient and can be easily identified since they are part of the unique mAb expressed by all lymphoma cells. The idea that Ids can be used to generate anti-idiotype (anti-Id) antibodies was initially proposed by Niels Jerne^20^. Since then, several anti-Id vaccines have been developed based on the use of anti-Id as surrogate antigens. Anti-Id vaccines have shown efficacy in both preclinical and clinical studies, being able to induce both humoral and cellular immune responses against tumor antigens^5^. Although their therapeutic efficacy has been generally modest, an anti-Id vaccine was approved in 2016 in Cuba and Argentina^21^. This vaccine, called Racotumomab (Vaxira), was raised against murine anti-ganglioside N-glycolyl (NGc) GM3 and has shown to be able to increase survival in non-small cell lung cancer patients.

While most anti-Id vaccines are based on mAbs, several studies have shown that polyclonal anti-Id vaccines could be more efficient, since they could function as surrogates of multiple epitopes from the same antigen^5^. In the case of B-cell lymphomas, Id vaccination could in principle stimulate polyclonal responses against the variable region of the Id, leading to potent cancer responses^4^.

As mentioned earlier, an important problem to generate Id vaccines against B-cell lymphomas is the production of sufficient amounts of the Id for immunization in a short period of time. In the present study we have developed a new and quick method to produce Ids based on the generation of mammalian cell lines using a noncytopathic self-replicative RNA vector derived from SFV (ncSFV). This vector encodes a puromycin resistance gene which allows for quick selection of transfected cells in the presence of that antibiotic. One of the main advantages of this system is that is fully dependent on cytoplasmic RNA replication, in contrast to more conventional methods of stable cell selection based on plasmid DNA integration. The latter approach is time-consuming and usually rely on the selection of high expressing clones^22^. We have previously shown that the ncSFV vector could be used to generate stable cells expressing human proteins with therapeutic potential, such as insulin-like growth factor I and cardiotrophin-1. Interestingly, these cell lines showed high stability, maintaining protein expression levels during at least ten passages, and with a very low mutation rate in the transgene^14^. In the present study we have applied the same technology to generate stable cell lines expressing the Id produced by mouse lymphoma A20.

Production of a full-length antibody requires expression of two different polypeptides in cells, i.e. the heavy (HC) and light chains (LC), which will assemble into a heterotetrameric protein. Several studies suggest that optimal expression of an antibody requires a ratio of LC:HC greater than one^18,23^, although the optimal ratio could depend on the cell line^23^. In order to obtain an optimal Id expression, we tested several vector configurations to express both HC and LC from the same ncSFV vector. The highest expression levels were obtained when we placed each antibody chain under independent SFV subgenomic promoters, especially if LC was upstream of HC (ncSFV-pac-A20sgPr). In this case, two different subgenomic RNAs will be produced, each one translating one of the mAb chains. This strategy has been previously used by us to express the two subunits of interleukin-12 (p35 and p40) from a cytopathic SFV vector, resulting in high expression levels of this heterodimeric cytokine^16^. The use of a 2A self-cleaving peptide placed between LC and HC led to slightly lower antibody levels, despite the fact that this strategy has been extensively used to express two proteins at similar levels^24^ and was succesfully used to express an antiPD-L1 mAb from a cytotopathic SFV vector^25^. It is possible that in our case the fact that the vector contains another 2A sequence between pac and HC, could decrease the total amount of LC released from the polyprotein, as suggested by the Western blot analysis shown in Supplementary Fig. 1. Interestingly, the use of an IRES element between LC and HC resulted in very low levels of HC, leading to low production of the antibody (Fig. 2 and Supplementary Fig. 1). This correlates with previous observations in which expression of the two interleukin-12 subunits separated by the same IRES, using an SFV vector, led to much lower expression of the cytokine compared to the use of independent subgenomic promoters^16^. Based on this initial characterization, a cell line selected with ncSFV-pac-A20sgPr vector was chosen for production and purification of A20 Id. The purified mAb showed a very similar size and glycosylation pattern compared to the one produced by hybridoma cells, which are also of mammalian origin (Fig. 4). Antibodies are usually highly N-glycosylated and this type of post-translational modification could affect their stability and immunogenicity^26^. In this regard, maintaining a mammalian glycosylation pattern could be an advantage of our system versus production methods based on insect cells or plants, although in the latter case targeted manipulation of plant N-glycosylation pathways, has enabled production of proteins with mammalian-like oligosaccharides^27^. The production yield of our system was not excessively high, reaching 1.6 mg/L/24 h after purification. However, given that in most clinical trials patients have been immunized with Id doses in the range of 0.5–2 mg^28^, our method would be efficient enough to generate the required amount of recombinant Id for each patient from a relatively low culture volume (1–2 L). Immunization of mice with A20 Id produced by the ncSFV system resulted in antitumor effects that were very similar to the ones observed in mice immunized with A20 produced by a hybridoma cell line, confirming that both Ids have similar properties and validating our system for production of Id vaccines.

An interesting alternative for protein-based Id vaccines is the use of genetic vaccines, which can be administered as DNA, mRNA, or viral vectors. In this type of vaccines, the antigen (Id) is expressed in vivo by transfected/infected cells, which could potentiate cellular immune responses, especially when these vectors are targeted to antigen presenting cells. This approach has been validated for B-cell lymphoma in a preclinical mouse model immunized with an adenovirus vector expressing A20 Id^6^ and will be tested in clinical trials against B-cell lymphomas using DNA vaccines encoding patient-specific Ids linked to immunostimulatory sequences^29,30^. Regarding mRNA vaccines, the recent success of Covid-19 vaccines based on mRNAs expresing the spike protein of SARS-CoV-2, makes this approach very interesting for Id vaccination. These vaccines rely on complexing the mRNA with cationic lipid nanoparticles that facilitate entry into cells. This approach could also be used for in vivo delivery of a self-replicating RNA expressing an Id, like the one used in this study, something that could increase its expression and stimulate innate immune responses, potentiating antitumor responses^31^.

## Supplementary Information

Supplementary Information.
