# Supplementary material for: Idiotype vaccines produced with a non-cytopathic alphavirus self-amplifying RNA vector induce antitumor responses in a murine model of B-cell lymphoma
Source: Sci Rep. 2021 Nov 2;11:21427. doi: 10.1038/s41598-021-00787-5 (PMC8563967; doi:10.1038/s41598-021-00787-5)

Idiotype vaccines produced with a non-cytopathic alphavirus self-amplifying RNA vector induce antitumor responses in a murine model of B-cell lymphoma

Erkuden Casales, Eva Martisova, Helena Villanueva, Ascensión López Díaz de Cerio, Susana Inoges, Noelia Silva-Pilipich, María Cristina Ballesteros-Briones, Alejandro Aranda, Jaione Bezunartea, Maurizio Bendandi, Fernando Pastor, Cristian Smerdou

Supplementary Figures

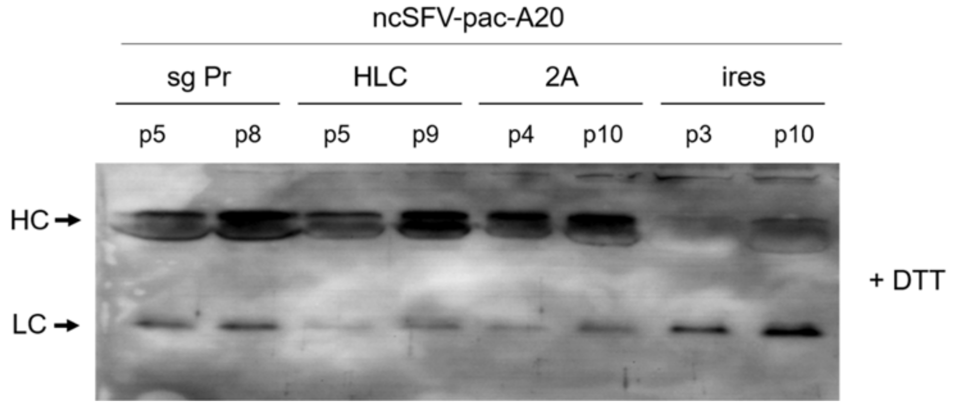

Supplementary Figure 1. Analysis of expression of HC and LC of recombinant A20 mAb in cell lines generated with ncSFV vectors. Supernatants from the indicated passages (p) of SFV-based stable cell lines generated as described in Fig. 1 were analyzed by Western blot with an anti-mouse IgG antibody under reducing conditions (+DTT).

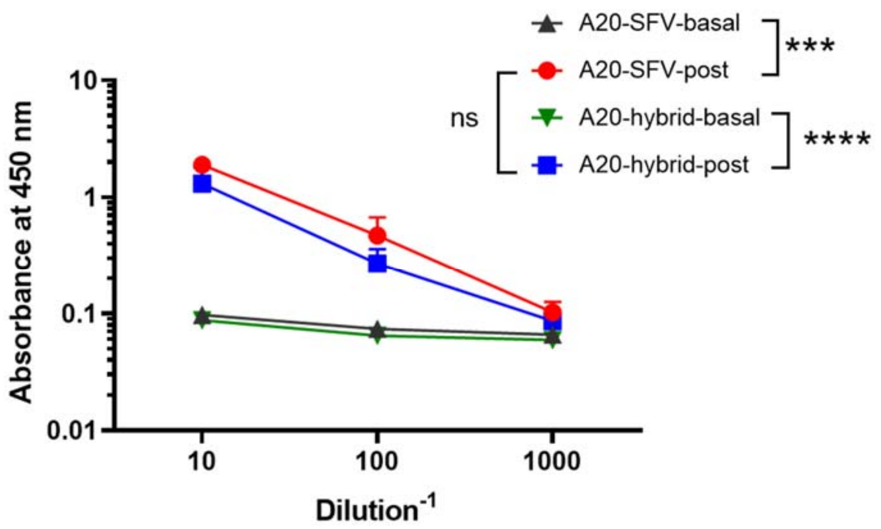

Supplementary Figure 2. Analysis of anti-idiotype antibodies in vaccinated mice. Sera from mice vaccinated with A20 mAb produced by ncSFV (A20-SFV) or hybridoma cells (A20-hybrid) were collected before the first immunization (basal) and ten days after the last vaccine boost (post). These sera were analyzed for their capacity to bind A20 mAb by ELISA as described in Materials and Methods. The graph was generated with Graphpad Prism software (version 9.2.0) and represents the mean ± SEM (n=5) of the absorbance obtained for the indicated sera dilutions. Data from one representative experiment out of three are shown. Statistical comparisons were performed for data obtained with the 1:10 dilution using ordinary one way ANOVA. ***, $p>0.001$; ****, $p<0.0001$, ns, non-significant.

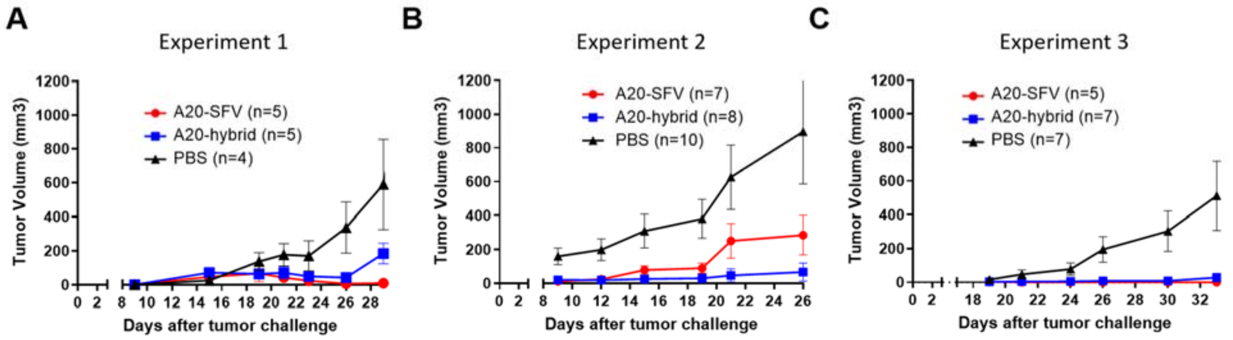

Supplementary Figure 3. Tumor growth in three independent experiments after vaccination of mice with purified A20. Balb/c mice were vaccinated with A20 mAb purified from the supernatant of the ncSFV-A20-sgPr cell line (A20-SFV) or from A20 hybridoma cells (A20-hybrid) conjugated to KLH and in combination with GM-CSF as indicated in the diagram shown in Figure 5. Control mice received PBS. Two weeks after the last immunization, mice were challenged with A20 tumor cells expressing A20 idiotype mAb and tumor growth in each treatment group is shown. Graphs A, B, and C correspond to three independent experiments performed with three different batches of A20 mAb. Data indicate mean ± SEM. Graphs were generated with Graphpad Prism software (version 9.2.0).

Supplementary Table 1. Oligonucleotides used to generate ncSFV-pac-A20 vectors

OLIGONUCLEOTIDE	SEQUENCE (5´-3´)
1	CCCGGGAAGTTGCCTGTTAGGCTG
2	CCAATCTAGGACCGCCGTAGAGGTTTAACACTCATTCCTGTTGAAGCT
3	ACCTCTACGGCGGTCCTAGATTGGTGCGTTAATACACAGAATTCTGATTGCACCATGGCATGGAACTTCATCATGGTC
4	CCCGGGCTATTTACCCGGAGTCCGGGAG
5	CCCGGGGCATGGAACTTCATCATGGTC
6	GTATTAACGCACCAATCTAGGACCGCCGTAGAGGTATCGATTTATTTACCCGGAGTCCGGGAGAAG
7	CGGTCCTAGATTGGTGCGTTAATACACAGAATTCTGATTGCACCATG*AAGTTGCCTGTTAGGCTG*TTG
8	CCCGGGCATATGTTAACACTCATTCCTGTTG
9	CTTCTCCCGGACTCCGGGTAAAAGAAGGCGCCGGAACTTCGACCTGCTGAAGCTGGCCGGCGAC
10	AACTTCGACCTGCTGAAGCTGGCCGGCGACGTGGAAAGCAACCCCGGACCGAAGTTGCCTGTTAGGCTGTTTGGTG
11	GTGAATTGATCCTTACTTAGTTAACACTCATTCCTGTTGAAG
12	GTTAACTAAGTAAGGATCAATTCACGCGTCGAGCATGCATCTAGG
13	AGTCGACCGCGGTTGTGGCAAGCTTATCATCG
14	ACCGCGGTCGACTGTATATCCATTTTCGGATCTGATCAGCACGTGATGGCATGGAACTTCATCATGGTC

Original, unprocessed gels shown in figures

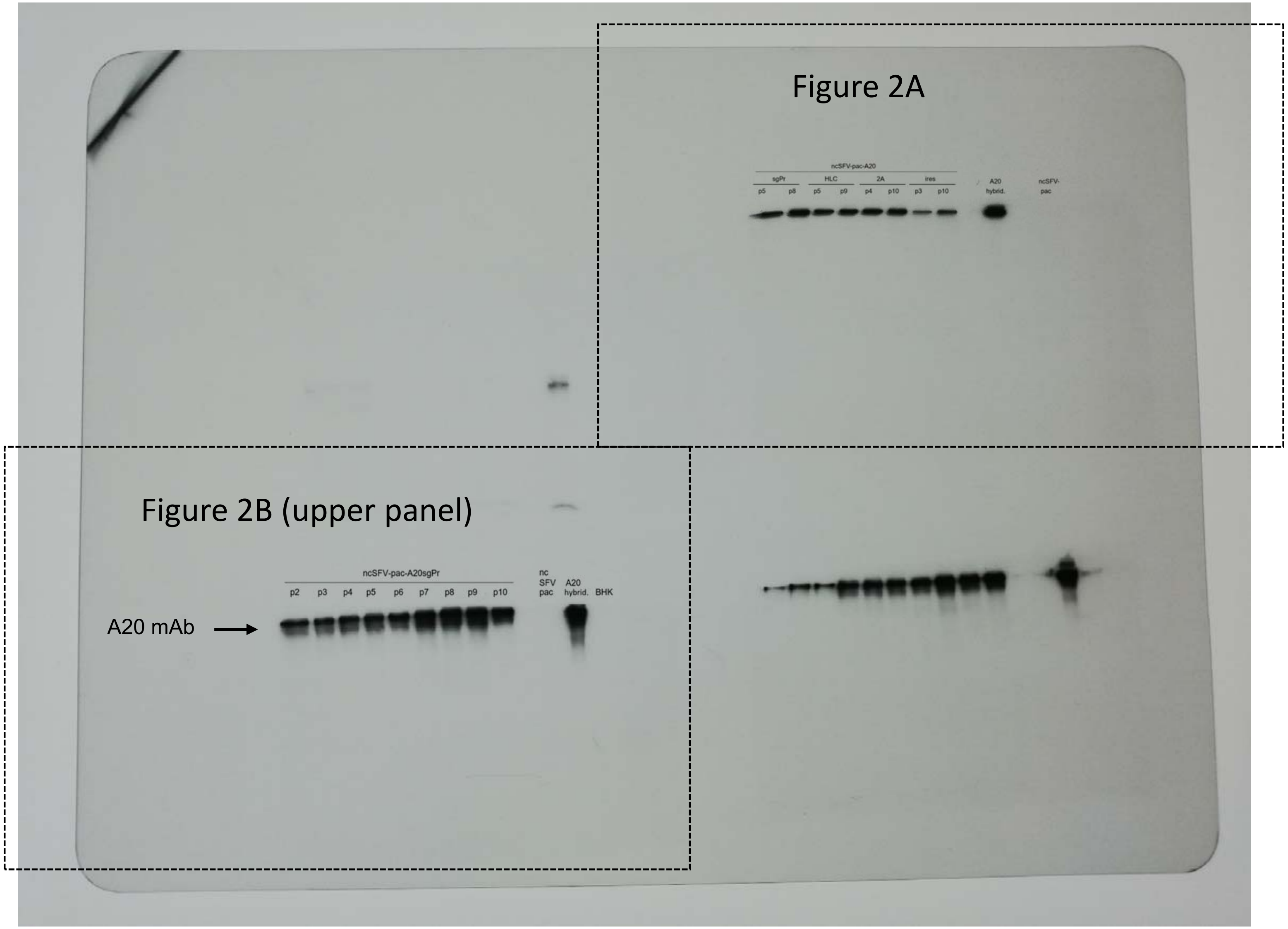

Figure 2B

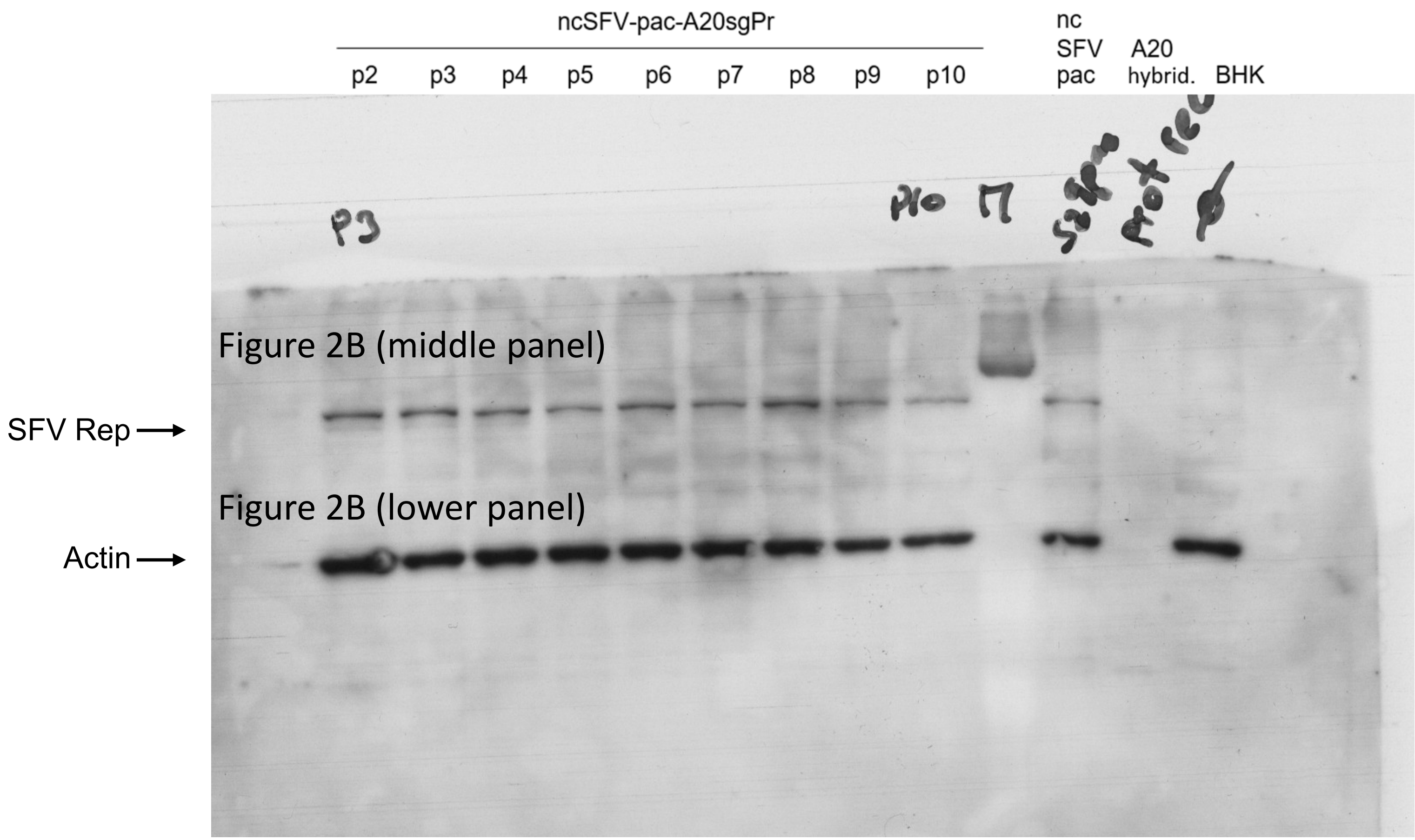
ncSFV-pac-A20sgPr
p2
p3
p4
p5
p6
p7
p8
p9
p10
nc SFV pac
A20 hybrid.
BHK
Figure 2B (middle panel)
SFV Rep
Figure 2B (lower panel)
Actin

Figure 3A
Upper panel

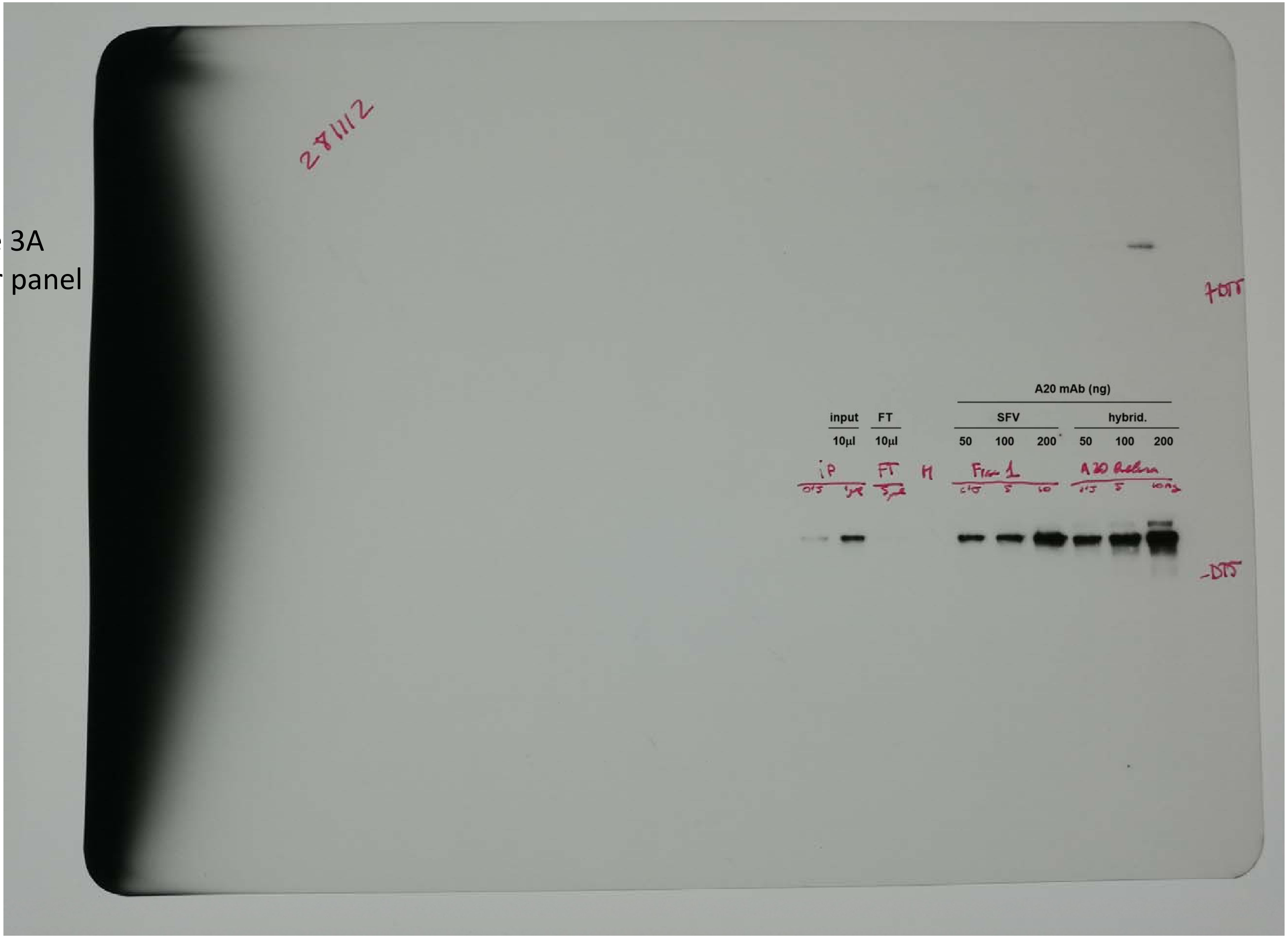

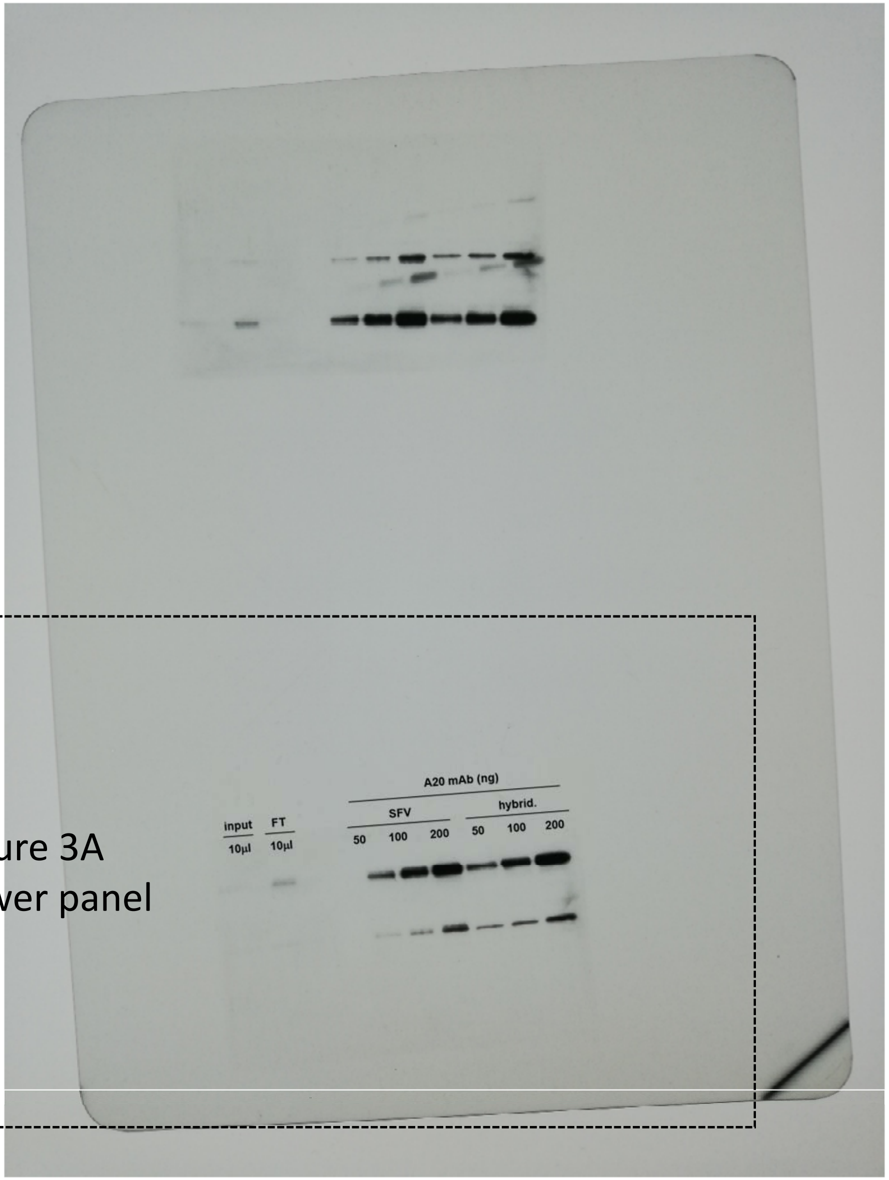

Figure 3A
Lower panel

Figure 3B
(upper gel)

Figure 3B
(lower gel)

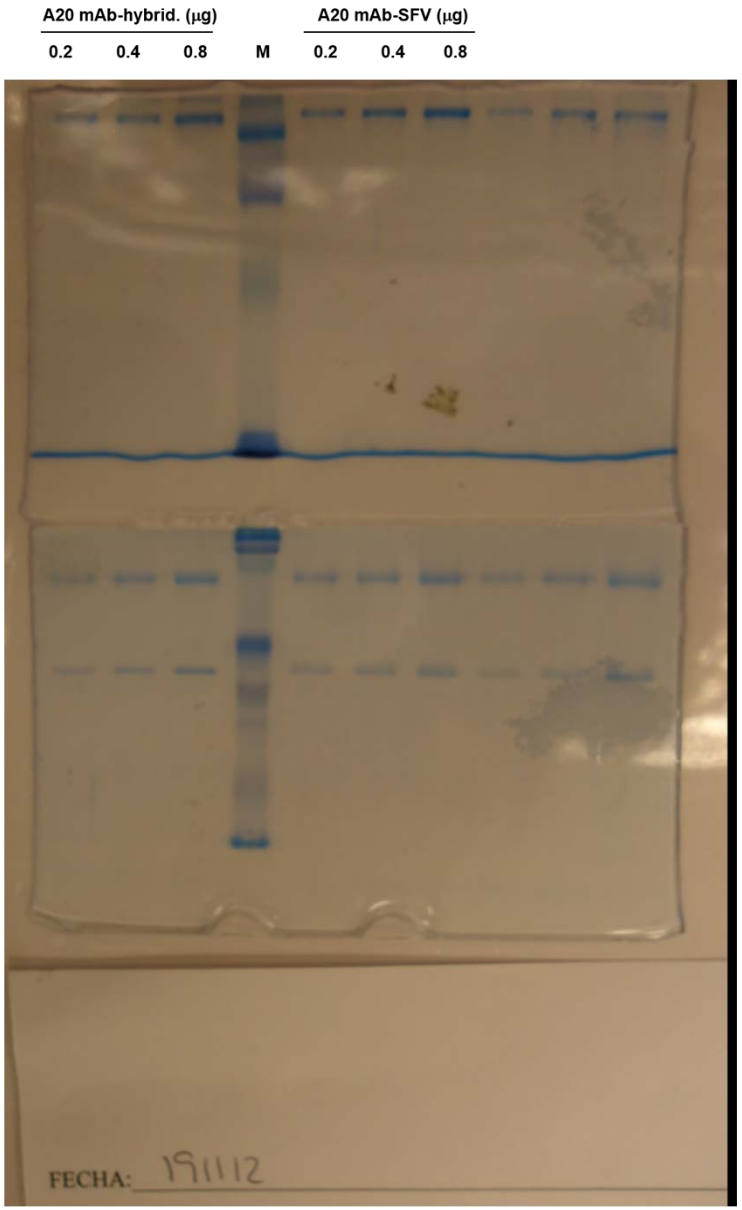

Figure 4

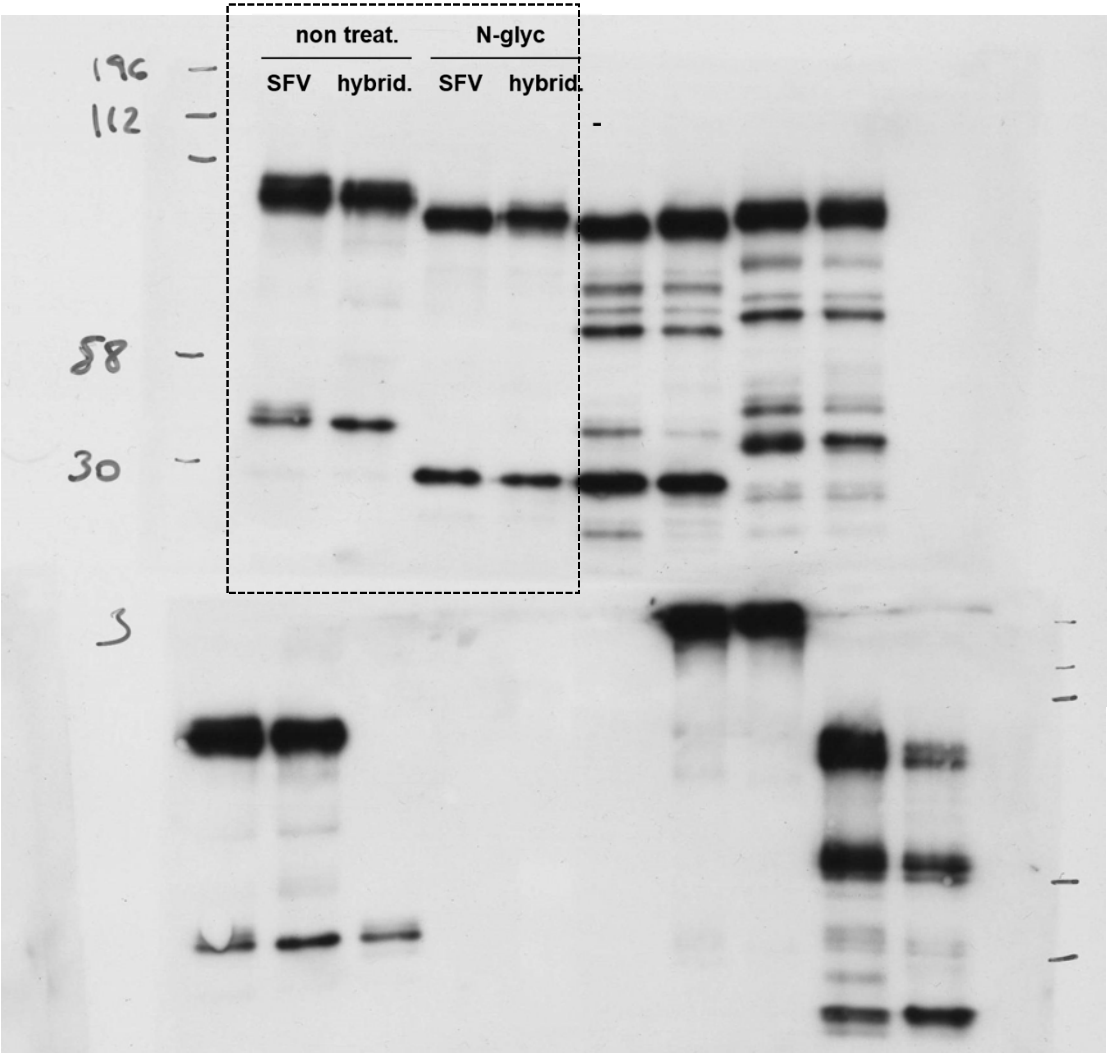

Figure S1

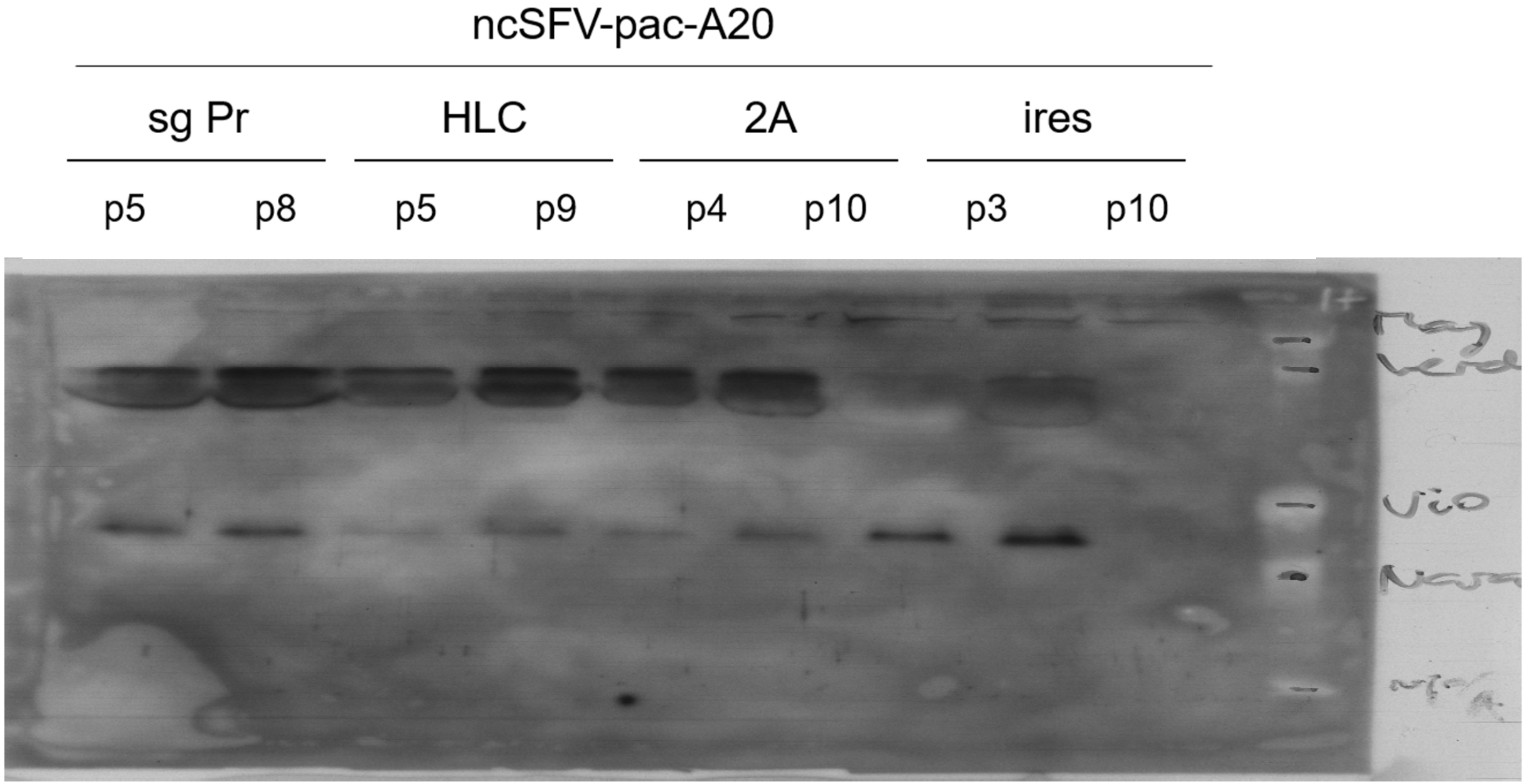

Supplement: Supplementary file 1 — Supplementary Information. [file 41598_2021_787_MOESM1_ESM.pdf]
